# Brain dysfunction and thyroid antibodies: autoimmune diagnosis and misdiagnosis

**DOI:** 10.1093/braincomms/fcaa233

**Published:** 2021-01-05

**Authors:** Cristina Valencia-Sanchez, Sean J Pittock, Carolyn Mead-Harvey, Divyanshu Dubey, Eoin P Flanagan, Sebastian Lopez-Chiriboga, Max R Trenerry, Nicholas L Zalewski, Anastasia Zekeridou, Andrew McKeon

**Affiliations:** 1 Department of Neurology, Mayo Clinic, Rochester, MN, USA; 2 Department of Laboratory Medicine and Pathology, Neuroimmunology Laboratory, Mayo Clinic, Rochester, MN, USA; 3 Health Science Research, Mayo Clinic, Scottsdale, AZ, USA; 4 Department of Neurology, Mayo Clinic, Jacksonville, FL, USA; 5 Department of Psychology, Mayo Clinic, Rochester, MN, USA; 6 Department of Neurology, Mayo Clinic, Scottsdale, AZ, USA

**Keywords:** Hashimoto encephalopathy, steroid-responsive encephalopathy associated with autoimmune thyroiditis, autoimmune encephalopathy

## Abstract

Hashimoto encephalopathy, also known as steroid-responsive encephalopathy associated with autoimmune thyroiditis, has been defined by sub-acute onset encephalopathy, with elevated thyroid antibodies, and immunotherapy responsiveness, in the absence of specific neural autoantibodies. We aimed to retrospectively review 144 cases referred with suspected Hashimoto encephalopathy over a 13-year period, and to determine the clinical utility of thyroid antibodies in the course of evaluation of those patients. One hundred and forty-four patients (all thyroid antibody positive) were included; 72% were women. Median age of symptom onset was 44.5 years (range, 10–87). After evaluation of Mayo Clinic, 39 patients (27%) were diagnosed with an autoimmune CNS disorder [autoimmune encephalopathy (36), dementia (2) or epilepsy (1)]. Three of those 39 patients had neural-IgGs detected (high glutamic acid decarboxylase-65, α-amino-3-hydroxy-5-methyl-4-isoxazolepropionic acid receptor-receptor and neural-restricted unclassified antibody), and 36 were seronegative. Diagnoses among the remaining 105 patients (73%) were functional neurological disorder (*n* = 20), neurodegenerative disorder (*n* = 18), subjective cognitive complaints (*n* = 14), chronic pain syndrome (*n* = 12), primary psychiatric (*n* = 11), sleep disorder (*n* = 10), genetic/developmental (*n* = 8), non-autoimmune seizure disorders (*n* = 2) and other (*n* = 10). More patients with autoimmune CNS disorders presented with sub-acute symptom onset (*P* < 0.001), seizures (*P* = 0.008), stroke-like episodes (*P* = 0.007), aphasia (*P* = 0.04) and ataxia (*P* = 0.02), and had a prior autoimmune history (*P* = 0.04). Abnormal brain MRI (*P* = 0.003), abnormal EEG (*P* = 0.007) and CSF inflammatory findings (*P* = 0.002) were also more frequent in the autoimmune CNS patients. Patients with an alternative diagnosis had more depressive symptoms (*P* = 0.008), anxiety (*P* = 0.003) and chronic pain (*P* = 0.002). Thyoperoxidase antibody titre was not different between the groups (median, 312.7 versus 259.4 IU/ml; *P* = 0.44; normal range, <9 IU/ml). None of the non-autoimmune group and all but three of the CNS autoimmune group (two with insidious dementia presentation, one with seizures only) fulfilled the autoimmune encephalopathy criteria proposed by Graus *et al.* (A clinical approach to diagnosis of autoimmune encephalitis. *Lancet Neurol* 2016; 15: 391–404.) (sensitivity, 92%; specificity, 100%). Among patients who received an immunotherapy trial at our institution and had objective post-treatment evaluations, the 16 responders with autoimmune CNS disorders more frequently had inflammatory CSF, compared to 12 non-responders, all eventually given an alternative diagnosis (*P* = 0.02). In total, 73% of the patients referred with suspected Hashimoto encephalopathy had an alternative non-immune-mediated diagnosis, and more than half had no evidence of a primary neurological disorder. Thyroid antibody prevalence is high in the general population, and does not support a diagnosis of autoimmune encephalopathy in the absence of objective neurological and CNS-specific immunological abnormalities. Thyroid antibody testing is of little value in the contemporary evaluation and diagnosis of autoimmune encephalopathies.

## Teaser

Valencia-Sanchez *et al.* reviewed the spectrum of cases referred to their Autoimmune Neurology Clinic with suspected Hashimoto encephalopathy, and found that almost three-quarters had an alternative non-autoimmune CNS diagnosis. They conclude that thyroid antibodies are of little utility in the evaluation of autoimmune encephalopathy in contemporary clinical practice.

## Graphical Abstract

**Graphical Abstract:**
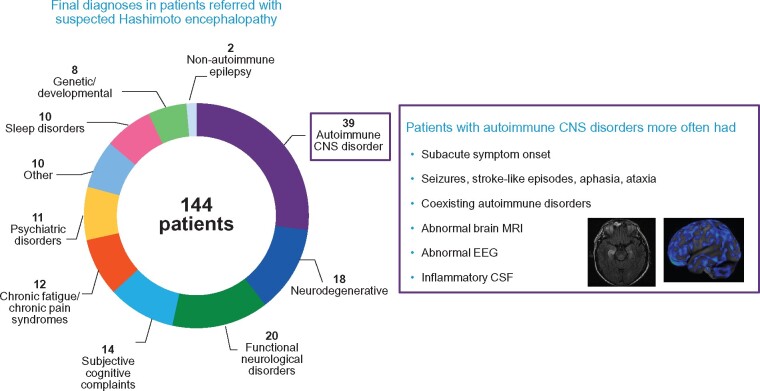

## Introduction

Hashimoto encephalopathy (HE) was first described in 1966 in a 49-year-old man who presented with stroke-like episodes and sub-acute encephalopathy months after the onset of Hashimoto thyroiditis (Brain *et al.*, 1966). Over the ensuing four decades, multiple cases were reported with various clinical findings (Shaw *et al.*, 1991; Chong *et al.*, 2003). An alternative moniker was proposed to encapsulate the concept of a triad of encephalopathy, thyroid autoimmunity (clinical, serological or both) and steroid-response [steroid-responsive encephalopathy associated with autoimmune thyroiditis (SREAT)] (Castillo *et al.*, 2006).

The majority of HE/SREAT cases are euthyroid or sub-clinically hypothyroid at presentation, but also have serological evidence to support the diagnosis of an autoimmune thyroid pre-disposition [thyroid peroxidase (TPO) and thyroglobulin antibodies]. By definition, patients present with encephalopathy, which is typically sub-acute and fluctuating. Additional reported features include seizures, psychiatric symptoms, myoclonus, tremor, transient aphasia and lateralized motor or sensory deficits (stroke-like episodes), sleep abnormalities and gait difficulties (Chong *et al.*, 2003; Castillo *et al.*, 2006; Ferracci and Carnevale, 2006; Schiess and Pardo, 2008; Laurent *et al.*, 2016; Litmeier *et al.*, 2016).

In our experience within our specialty referral practice, the diagnosis of HE/SREAT is assigned to a variety of patients with elevated thyroid antibody values and diverse neuropsychiatric symptomatic presentations, generally without objective demonstration of encephalopathy, CNS inflammation or objective steroid response. The reliance on subjective responses and outcomes appears to be pervasive, leading to over-diagnosis of autoimmune encephalopathy. Criteria for the diagnosis of HE were proposed in 2016, and was classified within the ‘probable’ autoimmune encephalitis (AE) category because the underlying pathogenic mechanism is unknown (Graus *et al.*, 2016).

Over the last 20 years, numerous neural IgG antibody biomarkers have been reported and incorporated into clinical testing profiles. Thus, some patients with thyroid autoimmunity are now better characterized in the course of neural IgG antibody testing (Mattozzi *et al.*, 2020). In addition, use of cognitive testing, imaging, EEG and generic CSF markers of inflammation has been promoted for objective case characterization (McKeon, 2016). A re-assessment of the value of thyroid antibodies as diagnostic markers in autoimmune encephalopathy seems timely.

Herein, we describe the spectrum of cases referred to the Autoimmune Neurology Clinic at Mayo Clinic, Rochester, Minnesota, with suspected HE/SREAT during the 13-year period since its inception.

## Materials and methods

### Inclusion criteria

This retrospective study was approved by the Mayo Clinic Institutional Review Board. The Advanced Cohort Explorer Data Retrieval tool was used to interrogate the electronic medical record so as to identify patients referred to the Autoimmune Neurology clinic for suspected HE/SREAT from 1 January 2006 to 1 August 2019. No patients from Mayo Clinic’s previous publication on the topic (which preceded the existence of our specialty clinic) were included (Castillo *et al.*, 2006). The search identified 171 patients, 27 were excluded because they did not sign the research consent (*n* = 11) or the final diagnosis was unclear due to incomplete evaluation (*n* = 16).

### Data ascertained from record review

Demographic and clinical characteristics, laboratory results including autoimmune serologic evaluation, CSF analysis, neuroimaging and EEG findings, and final clinical diagnoses were reviewed. MRI abnormalities were classified into normal/non-specific, suggestive of autoimmune aetiology, or other abnormalities. EEG findings were classified into normal/non-specific (mild background slowing with excess diffuse theta, or excess beta activity) or abnormal [moderate slowing (theta and occasionally delta frequencies), epileptiform abnormalities, rhythmic delta activity and triphasic waves] (Klass, 1981). PET findings were classified into normal or abnormal. Available results of diagnostic tests from outside our institution were collected. Due to the variability of TPO antibody reference ranges in different laboratories, quantitative results from our own institution alone were included, with the positivity threshold being >9 international units (IU)/l. Serum and CSF neural antibodies were tested at Mayo Clinic by standard clinical assays (indirect immunofluorescence assay, cell-based assay and immunoprecipitation assays as described in Supplementary Methods), and the data were recorded.

Data from cognitive testing ascertained during evaluation at Mayo Clinic were collected. Kokmen short test of mental status (STMS) was available in 121 patients (Kokmen *et al.*, 1991). The results of Neuropsychological test were available for 73 patients at the time of evaluation (10 were retested after immunotherapy). Details about Kokmen and neuropsychological cognitive domains results collected are available in Supplementary data.

### Case definitions, diagnostic criteria and treatment-response evaluation

The final diagnoses had been determined at the time of clinical evaluation by one or more of the authors in the Autoimmune Neurology Clinic after comprehensive clinical evaluation, testing and in some cases, after an immunotherapy trial confirmed objective improvement. Clinical characteristics and diagnostic test findings were compared between patients diagnosed with an autoimmune CNS disorder, and patients who were given an alternative diagnosis.

To compare Kokmen and neuropsychological testing scores, patients were classified into four groups according to final diagnosis: autoimmune CNS disorder, neurodegenerative disorder, primary non-neurological diagnosis [functional neurological disorder (FND), other psychiatric disorder, chronic pain syndrome, subjective cognitive complaints and sleep disorder] and other.

The sensitivity and specificity of the criteria for HE and other AE forms proposed by Graus *et al.* (2016) were evaluated utilizing a diagnosis of autoimmune CNS disorder in the setting of positivity for thyroid antibodies (but not neural IgGs) by one of the authors as gold standard. For HE criteria, we included the modification (adding sub-acute onset per the same authors’ revised HE criteria) (Mattozzi *et al.*, 2020): (i) sub-acute encephalopathy with seizures, myoclonus, hallucinations or stroke-like episodes; (ii) sub-clinical or mild overt thyroid disease; (iii) brain MRI normal or with non-specific abnormalities; (iv) the presence of serum thyroid antibodies (TPO and/or thyroglobulin); (v) the absence of well-characterized neuronal antibodies in serum and CSF and (vi) reasonable exclusion of alternative causes.

Among patients who received immunotherapy after our evaluation, characteristics were compared between patients who responded objectively, and those who did not. We only included in this analysis those patients who had objective baseline evaluations and post-treatment comparison, such as updated neurological examinations, neuropsychological testing, neuroimaging or EEG. Response to immunotherapy in epilepsy and encephalopathy 2 scores were also calculated (Dubey *et al.*, 2018).

### Statistical analyses

Categorical variables are presented as counts and percentages by group. Continuous variables are presented as median and range by group. Fisher exact tests were used to test the univariate association between clinical variable and diagnosis or treatment response group. Wilcoxon rank-sum tests were used to test univariate differences by diagnosis or treatment response group. The associations of autoimmune CNS diagnosis and treatment response with clinically relevant variables were quantified using univariate Firth’s penalized likelihood regression analysis. The associations were reported as odds ratio with 95% confidence intervals. Kokmen and neuropsychological testing scores were compared using Kruskal–Wallis tests. Where there were significant differences among the groups, *posthoc* pairwise differences were tested using the Dwass, Steel, Critchlow–Fligner Method, to control the familywise type I error. Hodges–Lehmann estimation method with Bonferroni adjustment for multiple testing was used to estimate adjusted confidence intervals for the median of differences between pairs of groups. All tests were two-sided and *P* values of <0.05 were considered statistically significant.

### Data availability

All collected data and statistical analysis are available for review.

## Results

### Demographic and background medical characteristics

The final study included 144 patients. One hundred and three (72%) were female. Median age at symptom onset was 44.5 years (range, 10–87). One hundred and two (71%) had autoimmune thyroid disease by history (Table 1). In brief, 8 of the 42 patients with no previously known thyroid disease were found to have sub-clinical hypothyroidism (elevated TSH with normal T4 and T3) at the time of our evaluation. One patient was diagnosed with Grave’s disease. Two additional patients developed sub-clinical hypothyroidism over the course of their follow-up at our institution. All patients by definition had previously documented positive thyroid antibodies, including TPO (140), thyroglobulin antibodies (35) or both (31).

**Table 1:** Demographic characteristics and clinical presentation of 144 patients referred for possible HE/SREAT diagnosis

	Autoimmune CNS disorder (*n* = 39)	Alternative diagnosis (*n* = 105)	Total (*n* = 144)	*P* value
Female	24 (61.5)	79 (75.2)	103 (71.5)	0.15
Median age (years) at onset (range)	46.0 (13–87)	44.0 (10–81)	44.5 (10–87)	0.08
Median duration (months) of symptoms (range)	16 (2–187)	30 (1–414)	25 (1–414)	**0.02**
Sub-acute onset (<3 months)	32 (82.1)	29 (27.6)	61 (42.4)	**<0.001**
Fluctuating course	23 (59)	46 (43.8)	69 (47.9)	0.13
History of autoimmune thyroid disease	24 (61.5)	78 (74.3)	102 (70.8)	0.15
Co-existing autoimmune disorder	13 (33.3)	17 (16.2)	30 (20.8)	**0.04**
Past history of neoplasm	4 (10.3)	5 (4.8)	9 (6.3)	0.25
Family history of autoimmune disorder	22 (56.4)	39 (37.1)	61 (42.4)	0.06
Prior immunotherapy	35 (89.7)	79 (75.2)	114 (79.2)	0.07
Reported immunotherapy response	33 (94.3)	29 (36.3)	62 (53.9)	**<0.001**
**Clinical presentation**				
Cognitive complaint	37 (94.9)	94 (89.5)	131 (91)	0.51
Seizures	10 (25.6)	8 (7.6)	18 (12.5)	**0.008**
Stroke-like episodes	8 (20.5)	5 (4.8)	13 (9)	**0.007**
Language deficit	11 (28.2)	13 (12.4)	24 (16.7)	**0.04**
Ataxia	6 (15.4)	4 (3.8)	10 (6.9)	**0.02**
Apraxia	3 (7.7)	7 (6.7)	10 (6.9)	>0.99
Motor or sensory deficits	15 (38.5)	22 (21)	37 (25.7)	0.05
Tremor	8 (20.5)	11 (10.5)	19 (13.2)	0.16
Myoclonus	2 (5.1)	5 (4.8)	7 (4.9)	>0.99
Hypersomnolence	5 (12.8)	17 (16.2)	22 (15.3)	0.8
Psychosis	13 (33.3)	21 (20)	34 (23.6)	0.12
Headache	9 (23.1)	27 (25.7)	36 (25)	0.83
Depression	5 (12.8)	37 (35.2)	42 (29.2)	**0.008**
Anxiety	1 (2.6)	25 (23.8)	26 (18.1)	**0.003**
Chronic pain	2 (5.1)	31 (29.5)	33 (22.9)	**0.002**

Categorical data provided as number (percentage). Bold values denote statistically significant results (*P* < 0.05).

After clinical and testing evaluations had been completed, 39/144 patients (27%) were assigned a diagnosis of an autoimmune CNS disorder (Table 2), and 105/144 (73%) were given an alternative clinical diagnosis. Alternative diagnoses included neurodegenerative disorder (*n* = 18), FND (*n* = 20), subjective cognitive complaints (*n* = 14), ≥1 of fibromyalgia, central sensitization, chronic pain syndrome or chronic fatigue (*n* = 12), primary psychiatric disorder (*n* = 11), sleep disorder in combination with other diagnosis (*n* = 10), other medical condition causing secondary encephalopathy (n = 10), behavioural or cognitive symptoms in patients with developmental or genetic disorders (*n* = 8) and epilepsy of non-autoimmune aetiology (*n* = 2). Specific diagnoses in each category are summarized in Table 3.

**Table 2:** Characteristics of patients diagnosed with autoimmune CNS disorder

No. Age/ sex	Onset and neurological presentation	MRI	CSF	EEG	PET metabolism	AE criteria
1. 30 M	<3 m Memory loss, behaviour change and hemiparesis (S-L)	-	–	–	SPECT: Global hypoperfusion	Prob HE
2. 53 F	<3 m Memory loss, aphasia, hemiparesis (S-L), loss of motivation and psychosis	T2-H frontal (B) Gad+	–	DS	NA	Poss AE
3. 48 F	<3 m Memory loss, disorientation and hypersomnia	–	OCB+	–	NA	Prob HE
4. 16 F	Unclear onset memory loss, hypersomnia, hallucinations and seizures	-	WBC 12	SW(T)	-	Prob HE^*^
5. 45 F	<3 m Confusion, hemiparesis and aphasia (S-L)	-	WBC 25	NA	NA	Prob HE
6. 41 M	<3 m Memory loss, confusion and behaviour change	-	–	NA	NA	Prob HE
7. 53 M	<3 m Memory, executive and visuospatial difficulties, behaviour change	-	–	-	Global hypo	Prob HE
8. 32 M	<3 m Confusion, hemiparesis and aphasia (S-L), combative	-	–	-	NA	Def AE
9. 50 M	<3 m Amnesia, aphasia (S-L), seizures, agitation, psychosis and hyper-religiosity	-	–	-	NA	Prob HE
10. 33 F	<3 m Memory, confusion, emotional lability, hallucinations and myoclonus	-	–	-	–	Prob HE
11. 44 M	<3 m Confusion, speech difficulty and facial weakness	-	WBC 15	-	–	Prob HE
12. 83 M	<3 m Confusion and hemiparesis (S-L)	-	–	-	NA	Prob HE
13. 26 F	<3 m Disorientation, psychosis and seizure	-	–	NA	NA	Prob HE
14. 63 M	<3 m Confusion, hemiparesis and aphasia (S-L)	-	–	DS	NA	Prob HE
15. 42 F	<3 m Confusion and hallucinations	-	–	–	NA	Prob HE
16. 13 M	<3 m Seizures and memory loss	-	–	SW(F)	NA	Prob HE
17. 67 F	<3 m Disorientation, confabulation, personality change, agitation and hallucinations	-	–	-	Focal (FT)hypo	Prob HE
18. 70 F	<3 m Disorientation and memory loss	-	–	–	NA	Def AE
19. 87 F	<3 m Disorientation, myoclonus and hypersomnia	-	–	SW(T)	Focal (FTP) hypo and hyper (T)	Prob HE
20. 42 F	<3 m Memory loss and hypersomnia	-	–	-	NA	Prob HE
21. 69 F	1–2 y memory loss 6 m more rapid decline	-	IgGs	–	Focal (PT) hypo	None^a^
22. 73 F	<3 m Confusion, behaviour change and seizures	Hippo atrophy & T2-H (R)	WBC 7	SZ	Focal (P) hypo and hyper (T)	Prob AE
23. 41 F	<3 m Confusion and emotional lability	-	–	–	NA	Prob HE
24. 30 F	6 m Seizures and memory loss	-	WBC 7	SW(P)	NA	Prob HE^*^
25. 43 M	<3 m Aphasia, memory loss, seizures and hemiparesis (S-L)	-	WBC 25 OCB+ IgGi	SW(FT), TIRDA	NA	Prob HE
26. 70 M	<3 m Memory loss, disorientation and seizures	T2-H temporal (B)	–	SZ (T)	–	Def LE
27. 56 M	<3 m Confusion, combative, hallucinations and cranial neuropathies (VII, IX, X)	-	–	NA	NA	Prob HE
28. 67 F	<3 m Confusion, behaviour change, mania and psychosis	-	–	DS	Focal (F)hypo	Prob HE
29. 51 F	<3 m Memory loss, psychosis and mania	-	–	–	–	Prob HE
30. 66 M	<3 m Confusion, agitation, hallucinations and ataxia	Frontal T2-H (R), Gad+ (dural)	WBC 8	GPEDs, DS	NA	Prob AE
31. 42 F	<3 m Confusion, delusions and seizures	Hippo atrophy and T2-H (L)	WBC 8	–	NA	Prob AE
32. 41 F	Months memory loss, hemiparesis and ataxia	-	OCB+	NA	Global hypo	Prob HE^*^
33. 44 F	<3 m Confusion, ataxia and dysarthria	Brainstem atrophy	IgGi	–	NA	Prob AE
34. 42 M	5-y Memory loss, aphasia, apraxia and tremor	Frontotemporal T2-H (L)	WBC 12 OCB+ IgGi	–	NA	None^a^
35. 68 F	<3 m Memory loss, aphasia, hallucinations and ataxia	-	–	SW(T), TIRDA	NA	Prob HE
36. 56 F	<3 m Memory loss, abnormal movements and right upper extremity	Hippo atrophy & T2-H(B), Gad+	–	SW (T)	NA	Def LE
37. 62 M	4 m Memory loss, concentration and neuropathy	–	–	–	NA	Prob HE^*^
38. 39 F	<3 m Multiple cranial neuropathies	-	–	NA	-	Prob HE
39. 61 F	8 m New musicogenic seizures	-	–	SW(T), SZ (FT)	NA	None^b^

+ yes, - normal or non-specific, S-L, stroke-like; m, months; y, years; T2-H, T2-hyperintensity; AE, autoimmune encephalitis; HE, Hashimoto encephalopathy; LE, limbic encephalitis; DS, diffuse slowing; SW, sharp waves; SZ, seizure; TIRDA, temporal intermittent rhythmic delta activity; GPEDs, generalized periodic epileptiform discharges; M, male; F, female; NA, not available; Hippo, hippocampus; F, frontal; T, temporal; P, parietal; FT, fronto-temporal; FTP, fronto-temporo-parietal; PT, parieto-temporal; L, left; R, right; B, bilateral; Gad+, contrast enhancement; IgGi, elevated IgG index; IgGs, elevated IgG synthesis rate; Prob, probable; Poss, possible; Def, definite.

a Autoimmune dementia.

b Autoimmune epilepsy.

* Symptom onset excluded.

**Table 3:** Alternative diagnoses among 105 patients referred with suspected HE/SREAT

Alternative clinical diagnoses	*N* (%)
Neurodegenerative disorder	18 (17.1%)
Alzheimer disease (5)	
Fronto-temporal dementia (1)	
Primary progressive aphasia (2)	
Lewy body dementia (2)	
Posterior cortical atrophy (1)	
Vascular dementia (1)	
Probable Creutzfeldt–Jakob disease (1)	
Other neurodegenerative disorder (5)	
Functional neurological disorder	20 (19%)
Subjective cognitive complaints	14 (13.3%)
Fibromyalgia/chronic fatigue/chronic pain syndromes	12 (11.4%)
Psychiatric disorder	11 (10.5%)
Depression (3)	
Generalized anxiety disorder (2)	
Bipolar disorder (2)	
Obsessive compulsive disorder (1)	
Schizoaffective disorder (1)	
Schizophreniform disorder (2)	
Sleep disorder, in combination with other diagnosis	10 (9.5%)
Obstructive sleep apnoea + functional (3)	
Obstructive sleep apnoea + fibromyalgia + depression (2)	
Obstructive sleep apnoea + anxiety (1)	
Obstructive sleep apnoea + narcolepsy (1)	
Primary hipersomnia + functional tremor + fibromyalgia (1)	
Insomnia + functional tremor + depression (1)	
Insomnia +subjective cognitive (1)	
Other medical condition	10 (9.5%)
Severe hypothyroidism (2)	
Radiation leucoencephalopathy (1)	
Post-surgical movement disorder (1)	
Side effects antiepileptic drugs (1)	
Mast cell activation disorder (1)	
Static encephalopathy after intracranial haemorrhage + status epilepticus (1)	
Intracranial hypotension (1)	
Leucoencephalopathy (toxic/vascular) (1)	
Central pontine myelinolysis + functional tremor and spells (1)	
Genetic/developmental disorder with behavioural/cognitive symptoms	8 (7.6%)
Trisomy 21 (1)	
Trisomy 2 (1)	
Mitochondrial cytopathy (1)	
Adult onset neuronal intra-nuclear inclusion disease (1)	
Autism spectrum disorder (1)	
Other developmental disorder (3)	
Epilepsy	2 (1.9%)
Idiopathic focal epilepsy (1)	
Focal epilepsy secondary to meningioma (1)	

### Sensitivity and specificity of diagnostic criteria for autoimmune encephalopathy and Hashimoto encephalopathy

None of 105 patients with alternative diagnoses met any of the AE diagnostic criteria (Graus *et al.*, 2016) (specificity, 100%). With omission of the ‘exclusion of an alternative condition’ criterion, 10 patients with an alternative diagnosis met the other five HE criteria (specificity, 90%). Of 39 patients with an autoimmune CNS disorder, all but three fulfilled diagnostic criteria for one or other diagnosis as previously suggested by others (Graus *et al.*, 2016) [probable HE, 27 (13 of whom also fulfilled criteria for possible AE); probable AE, 4; definite AE, 2; definite limbic encephalitis, 2; and possible AE, 1], sensitivity 92%. In the context of this retrospective review, patients with a sub-acute onset and course, but for whom precise symptom duration was not documented (should be <3 months), were included. The three remaining patients did not meet those criteria but were classifiable as autoimmune dementia (2) or autoimmune epilepsy (1), based on prior publications (Flanagan *et al.*, 2010; Quek *et al.*, 2012). The autoimmune dementia patients (21 and 34, Table 2) had cognitive presentations without delirium typical of encephalopathy, and an onset reported as insidious. Both patients had inflammatory CSF and immunotherapy response. The autoimmune epilepsy patient (39, Table 2) had a seizure disorder alone, normal neuropsychometric testing, brain MRI and CSF, though had glutamic acid decarboxylase 65-kD isoform (GAD65) antibody detected at high titre.

### Prior history

More patients in the group diagnosed with autoimmune CNS disorders had history of other autoimmune disorders (33% versus 16%; *P* = 0.04), abnormally low vitamin B12 (23% versus 1%; *P* = 0.001). Co-existing autoimmune disorders in both groups included pernicious anaemia (*n* = 5), coeliac disease (*n* = 4), psoriasis (*n* = 2), vitiligo (*n* = 4), asthma (*n* = 4), Sjogren’s syndrome (*n* = 3), lupus (*n* = 2), rheumatoid arthritis (*n* = 2), type-1 diabetes mellitus (*n* = 2) and one each for Crohn’s disease, ulcerative colitis, sclerosing cholangitis, granulomatosis with polyangiitis, alopecia areata, autoimmune dermatological disorder, multiple sclerosis and prior Guillain–Barre syndrome.

More patients in the group diagnosed with autoimmune CNS disorders had family histories of autoimmune disorders, and personal history of cancer, although the differences were not statistically significant (56% versus 37%; *P* = 0.06 and 10% versus 5%; *P* = 0.25, respectively).

### Clinical presentation

Patients with autoimmune CNS disorders were more likely to have sub-acute (<3 months) onset (82% versus 28% *P* < 0.001). The median duration of the symptoms prior to evaluation in our clinic was shorter for patients with an autoimmune CNS diagnosis (16 versus 30 months, *P* = 0.02).

More patients diagnosed with autoimmune CNS disorders presented with objective language deficit (28% versus 12% *P* = 0.04), seizures (26% versus 8% *P* = 0.008), stroke-like episodes (21% versus 5% *P* = 0.007) and ataxia (15% versus 4% *P* = 0.02). More patients with an alternative diagnosis presented with depressive symptoms (35% versus 13%, *P* = 0.008), anxiety (24% versus 3%, *P* = 0.003) and chronic pain (30% versus 5%, *P* = 0.002) (Table 1). Odds ratio for these clinical variables from univariate Firth’s regression analysis are listed in Supplementary Table 1.

Of the eight patients in the autoimmune CNS group with stroke-like episodes (defined as acute onset and transient unilateral limb weakness with or without facial weakness, and/or language deficit), four presented with hemiparesis, three with hemiparesis and language deficit, and one with language deficit only. Three patients had recurrent episodes, and in one of them the hemiparesis was alternating. All had encephalopathy accompanying those episodes. Of the five patients in the alternative diagnosis group with stroke-like episodes, one was eventually diagnosed with adult-onset intra-nuclear inclusion disease (episodic hemiparesis and neglect), and four had FND with the absence of encephalopathy, functional signs on exam (psychogenic non-epileptic spells, functional hemisensory loss and functional gait) and normal para-clinical diagnostic results.

Patients diagnosed with FND presented with functional neurological symptoms and signs including psychogenic non-epileptic spells captured with normal EEG (*n* = 7), functional gait disorders (*n* = 7), movement disorder (*n* = 6), hemiparesis (*n* = 4), speech disorders (*n* = 4), chronic subjective dizziness (*n* = 2), sensory loss (*n* = 1), convergence spasm (*n* = 1) and dissociative amnesia (*n* = 1).

### Cognitive testing

In the autoimmune CNS disorder group, 28/33 patients with available Kokmen scores, and 15/20 patients with available neuropsychological testing, had received immunotherapy prior to their first evaluation at Mayo Clinic.

There were no significant differences in the total Kokmen scores between the autoimmune CNS disorder group (*n* = 33) and ‘non-neurological diagnosis’ (*n* = 58) groups (*P* = 0.16). There was a significant difference in the recall sub-scale (*P* = 0.03), with the autoimmune CNS disorder group having a lower score (Supplementary Table 2).

On neuropsychological testing, there were significant differences between the autoimmune CNS disorder (*n* = 20) and non-neurological (*n* = 36) groups in the Auditory Verbal Learning Test delayed recall score (*P* = 0.02), with the autoimmune CNS disorder group having a lower score (Supplementary Table 3). In the non-neurological group (*n* = 36), although some of the patients had abnormal testing scores (*n* = 14), the interpretation of the results by the clinical neuropsychologist provided context for these abnormalities. In the FND group (*n* = 8), one patient had inconsistencies, two had variable attention and one had mild impairment of verbal learning. In the chronic pain syndrome group (*n* = 6): one patient demonstrated inconsistent scores within the same testing domains (suggestive of a non-neurological illness), one mild inefficiencies, one mild executive dysfunction and one suggested lifelong learning disability. Among the patients with other psychiatric diagnosis (*n* = 6), two had mild dysfunction attributed to depression and one had abnormal memory and executive function. Among the patients with sleep disorders (*n* = 7), one had inconsistencies, one mild attention and speed deficits and one perseveration.

Additionally, the examining neurologist also commented on inconsistencies in seven patients eventually diagnosed with FND, such as demonstrating superior cognitive function in the course of giving the medical history followed by disproportionate difficulty with cognitive testing (*n* = 4), or cogently critiquing the neurologist’s diagnosis at the follow-up visit (*n* = 3).

There were significant differences between the autoimmune CNS disorder (*n* = 33) and neurodegenerative (*n* = 16) groups in the total and subscale Kokmen scores, with patients in the autoimmune CNS group having higher scores (Supplementary Table 2). On neuropsychological testing, there were significant differences in the Wechsler Adult Intelligence Scale perceptual organization score (*P* = 0.02), with lower scores in the neurodegenerative group (*n* = 9). The Trail Making Test part *B* score was also different between these groups (*P* = 0.03), with higher scores in the neurodegenerative group (Supplementary Table 3).

### Para-clinical diagnostic test results

Imaging, EEG and laboratory findings for all patients are summarized in Table 4. Median TPO antibody titre in patients with an autoimmune CNS disorder [312.7 IU/ml (range, 14.4–950)] was not significantly different from those with an alternative diagnosis [259.4 IU/ml (range, 9.9–950)], *P* = 0.94. The number of patients with TPO antibody values previously reported as ‘high titre’ (>200 IU/ml) was not different among the groups (41% versus 37% *P* = 0.82) (Mattozzi *et al.*, 2020).

**Table 4:** Summary of the diagnostic testing results in patients with autoimmune CNS disorders and alternative diagnosis

Diagnostic test	Autoimmune CNS disorder (*n* = 39)	Alternative diagnosis (*n* = 105)	Total (*n* = 144)	*P* value
Median TPO titre IU/ml (range)^a^	312.7	259.4	271.5	0.44
	(14.4–950)	(9.9–950)	(9.9–950)	
	(*N* = 22)	(*N* = 75)	(*N* = 97)	
Low vitamin B12	6/26 (23.1)	1/70 (1.4)	7 (7.3)	**0.001**
Non-neural antibodies	6/34 (17.6)	17/90 (18.9)	23/124 (18.5)	>0.99
Neural antibodies in serum	14/39 (35.9)	21/101 (20.8)	35/140 (25)	0.08
MRI abnormalities suggesting AE	8 (20.5)	4/104 (3.8)	12/143 (8.4)	**0.003**
Abnormal EEG	14/33 (42.4)	14/85 (16.5)	28/118 (23.7)	**0.007**
Abnormal PET	8/14 (57.1)	18/42 (42.9)	26/56 (46.4)	0.37
CSF inflammatory^b^	20/39 (51.3)	20/88 (22.7)	40/127 (31.5)	**0.002**
WBCs, >5 cells/μl	9/39 (23.1)	1/88 (1.1)^c^	10/127 (7.9)	**<0.001**
Protein level, >50 mg/dl	16/39 (41)	20/88(22.7)	36/127 (28.3)	0.05
Positive OCB	4/30 (13.3)	0/81 (0)	4/111 (3.6)	**0.005**
Elevated IgG index and synthesis rate	3/29 (10.3)	0/81(0)	3/110 (2.8)	**0.017**

Categorical data is provided as number (percentage). Bold values denote statistically significant results (*P* < 0.05).

TPO, thyoperoxidase antibody; WBC, white blood cells; OCB, oligoclonal bands.

a Patients with abnormal (>9) value at Mayo Clinic.

b CSF inflammatory: WBC, >5 cells/μl, positive OCBs, high IgG index or synthesis rate.

c This patient had adult-onset neuronal intra-nuclear inclusion disease.

Patients with autoimmune CNS disorders were more likely to have abnormalities on MRI supportive of autoimmune encephalopathy (21% versus 4% *P* = 0.003) including temporal T2-hyperintensity unilateral (*n* = 3) or bilateral (*n* = 2), frontal T2-hyperintense signal with parenchymal (*n* = 1) or dural (*n* = 1) contrast enhancement, and brainstem atrophy (*n* = 1). Patients with autoimmune CNS disorders were also more likely to have an abnormal EEG (42% versus 17% *P* = 0.007).

**Figure 1:**
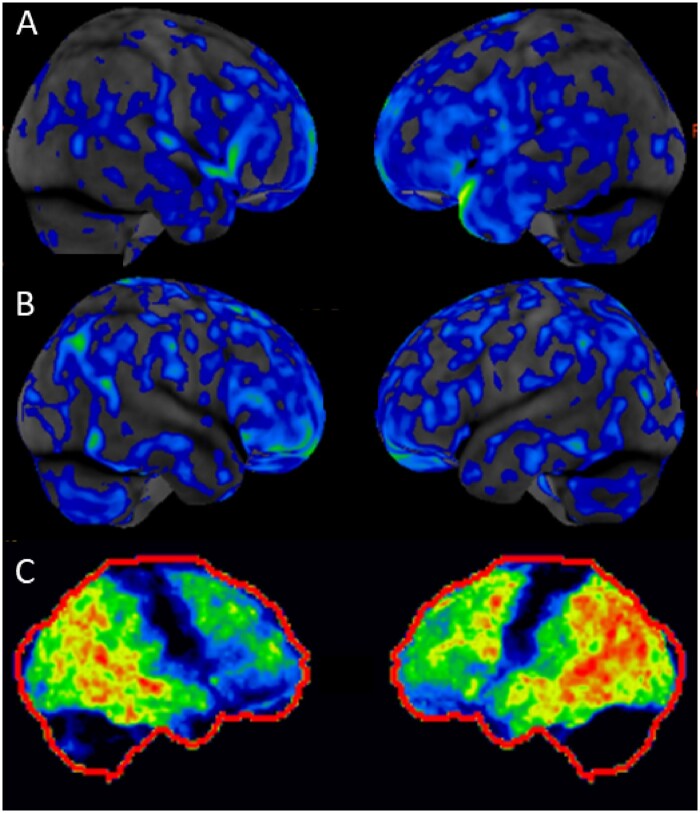
**Representative FDG-PET scan findings in two patients diagnosed with autoimmune encephalopathy and one patient with probable neurodegenerative disorder.** FDG-PET images, lateral views. (**A**) Diffuse hypometabolism (patient 32, Table 2). (**B**) Frontal and temporal hypometabolism (patient 17, Table 2). (**C**) Profound temporal and parietal hypometabolism and mild frontal hypometabolism (patient 45, Table 6).

Fifty-six patients had functional brain imaging, FDG-PET (55) and SPECT (1). Abnormal findings were reported in 8/14 (57.1%) patients in the autoimmune CNS group, and 18/42 (42.9%) with an alternative diagnosis. The abnormal findings in patients with autoimmune CNS disorders included global hypoperfusion on SPECT (*n* = 1), and on FDG-PET, global hypometabolism (*n* = 2), focal hypometabolism (*n* = 3, mild frontotemporal, bilateral frontal and moderate parietotemporal) and mixed hypometabolism and hypermetabolism (*n* = 2, mild frontotemporoparietal hypometabolism with temporal hypermetabolism, 1; bilateral parietal hypometabolism with temporal hypermetabolism, 1) (Fig. 1). In the group with an alternative diagnosis, 11 patients had a typical FDG-PET dementia pattern, and all of them were eventually diagnosed with a neurodegenerative disorder [Alzheimer’s disease (*n* = 4), frontotemporal dementia (*n* = 1), Lewy body dementia (*n* = 1), primary progressive aphasia (*n* = 1), posterior cortical atrophy (*n* = 1) and other neurodegenerative disorder (*n* = 3)].

In CSF studies, patients with autoimmune CNS disorders were more likely to have inflammatory CSF, defined as the detection of at least one of: elevated white blood cell (WBC) count, CSF-exclusive oligoclonal bands (OCB) or elevated IgG index/synthesis rate (51.3% versus 22.7%, *P* = 0.002). Protein level of >50 mg/dL was not significantly different in frequency between the groups (41% versus 23%, *P* = 0.05).

Odds ratio for the diagnostic test results associated with an autoimmune CNS diagnosis by univariate Firth’s regression analysis are listed in Supplementary Table 1.

In the autoimmune CNS disorder group alone, three patients were found to have encephalopathy-specific neural antibodies including high titre GAD65 (serum only tested), α-amino-3-hydroxy-5-methyl-4-isoxazolepropionic acid receptor (AMPA-R) antibody and unclassified antibody (which robustly stained murine brain on immunofluorescence assay, but no other tissues [gut and kidney], in both serum and CSF). None of the serum or CSF specimens from patients with non-autoimmune CNS disorders demonstrated specific binding to brain tissue on immunofluorescence assay. Low-titres of other neural antibodies, less specific for neurological autoimmunity were found in patients of both groups (*P* = 0.08) (Supplementary Table 4).

### Treatment responders versus non-responders

In total, 110 of 144 patients had received an immunotherapy trial at an outside facility prior to evaluation at Mayo Clinic (76%). The patients diagnosed with an autoimmune CNS disorder more frequently reported clinical improvement at initial consultation than patients ultimately given an alternative diagnosis (94% versus 36%, *P* < 0.001); objective data was generally not available from outside physicians.

After the evaluation at Mayo Clinic, 24 patients in the autoimmune CNS disorders group were recommended to undertake an additional immunotherapy trial. Of the remaining 15 patients, 8 had remitted and discontinued treatment and 7 were advised to continue treatment already initiated. Objective testing post-treatment demonstrating improvement was available in 16 patients (Table 5). Treatments were intravenous methylprednisolone (IVMTP) alone (13), IVMTP and plasma exchange or intravenous immunoglobulin (IVIg) (2) and IVIg alone (1). Seven of nine patients with Kokmen scores before and after immunotherapy available demonstrated improvement. In one patient, the Kokmen score after treatment was 4 points lower but neuropsychological testing showed improvement. One score (38/38) did not change because the patient already had cognitive recovery after a prior steroid course, but had persisting seizures which improved after IVMTP (patient 4, Table 5). Neuropsychological testing demonstrated post-treatment improvement in five patients (median time follow-up testing 3.5 months; range, 1–6). An example of the neuroimaging findings of two patients who experienced improvement in MRI and SPECT abnormalities is shown in Supplementary Fig. 1. In one patient, with elevated CSF OCB detected, although Kokmen score improved 5 points, PET hypometabolism pattern was unchanged (patient 32, Table 5). EEG demonstrated improvement of abnormalities in three patients. In one patient, although EEG did not significantly change after treatment, her neurological exam (ataxia and aphasia) and Kokmen score improved (patient 35, Table 5). Four of the patients reported complete recovery after immunotherapy. The median follow-up after onset of symptoms was 3.4 years (range, 0.4–12.8).

**Table 5:** Clinical and diagnostic tests findings, among autoimmune CNS treatment responders

No Age/ sex	Clinical presentation	Abnormal testing	Cognitive testing	Prior IT and outcome	IT trial at Mayo	Measurements improved after IT	Additional IT	Relapses off IT	Follow-up and mRS
1 30 M	Memory loss, behaviour change and hemiparesis (S-L)	PET	STMS 31 NPS abnormal	None	IVMTP	Frequency S-L STMS 31→35 SPECT	MTX (SE) MMF (SE) AZT (SE) >7 y	Yes	12.8 y mRs 1
2 53 F	Memory loss, aphasia, hemiparesis (S-L), loss of motivation and psychosis	MRI EEG	STMS 29	IVMTP and pred Improved	IVMTP and PLEX	STMS 23→31 MRI	Pred and MMF 2 y	Yes	2.6 y mRs 4
4 16 F	Memory loss, hypersomnia, hallucinations and seizures	CSF EEF	STMS 38 NPS normal	Pred, PLEX, IVIG Improved	IVMTP	Seizure frequency EEG	Pred 6 m MMF 2 y	Yes	9.2 y mRs 2
7 53 M	Memory, executive and visuospatial difficulties and behaviour change	PET	STMS 38 NPS abnormal	IVMTP Improved	IVIG	NPS	IVIG 16 m MMF 8 y	Yes	9.8 y mRs1
17 67F	Disorientation, confabulation, personality change, agitation and hallucinations	PET	STMS 31 NPS abnormal	IVMTP, IVIG, PLEX, pred Improved	IVIG and IVMTP	Exam, confusion, agitation and confabulation	Pred and MMF initiated	Yes	2.4 y mRs 4
19 87 F	Disorientation, myoclonus and hypersomnia	EEG PET	STMS 19	None	IVMTP	STMS 19→34	IVMTP 3 m and MMF initiated	No	0.4y mRs 2
20 42 F	Memory loss and hypersomnia	–	STMS 36 NPS abnormal	None	IVMTP	STMS 36→38 NPS	IVMTP 4 m IVIG 2 m (SE) AZT 5 m (SE)	Yes	3.5 y mRs 2
21 69 F	Memory loss	CSF	STMS 18 NPS abnormal	Pred Improved	IVMTP	STMS 18→14 NPS	AZT initiated	Yes	1.8 y mRs 3
22 73 F	Confusion, behaviour change and seizures	MRI CSF EEG PET	STMS 29 NPS abnormal	Pred, IVMTP Improved	IVMTP	STMS 29→36 NPS EEG	IVMTP 10 m	Yes	1.8 y mRs 0
24 30 F	Seizures and memory loss	CSF EEG	STMS 34 NPS abnormal	None	IVMTP	Seizure frequency EEG	IVMTP 3 m and MMF 10 m Relapse—IVMTP 3 m & resume MMF	Yes	3.2 y mRs1
27 56M	Confusion, combative, hallucinations and cranial neuropathies (VII, IX, X)	–	NA	IVMTP, PLEX, pred Improved	IVMTP	Cranial neuropathies and headache	AZT 1 y (SE)	Yes	2.5 y mRs 1
28 67 F	Confusion, behaviour change, mania and psychosis	EEG PET	STMS 35 NPS abnormal	Pred Improved	IVMTP	NPS	MMF initiated	Yes	4.4 y mRs2
30 66M	Confusion, agitation, hallucinations and ataxia	MRI CSF EEG	NA	IVIG PLEX	IVMTP Pred	Exam, hallucinations and encephalopathy	None	No	1.4y mRs 0
32 41 F	Memory, hemiparesis, ataxia	CSF PET	STMS 19	IVMTP, AZT, MMF, RTX Improved	IVMTP	STMS 19→24 PET	RTX continued	Yes	6.1 y mRs 2
35 68 F	Memory loss, aphasia, hallucinations and ataxia	EEG	STMS 31	None	IVMTP	Ataxia and aphasia STMS 31→36	None	No	5.9y mRs2
38 39F	Multiple cranial neuropathies	–	NA	Pred, IVIG Improved	IVMTP	Cranial neuropathies	AZT 3 y IVMTP for relapse CYC initiated	Yes (on AZT)	12.2y mRs1

IT, immunotherapy; STMS, Kokmen short test of mental status; NPS, neuropsychological testing; mRs, modified Rankin score; M, male; F, female; NA, not available; Pred, prednisone; IVMTP, intravenous methylprednisolone; IVIG, intravenous immunoglobulin; PLEX, plasma exchange; MMF, mofetil mycophenolate; AZT, azathioprine; RTX, rituximab; CYC, cyclophosphamide; w, week; m, month; y, year; SE, side effects.

Twelve patients in the alternative diagnosis group received an immunotherapy trial, seven of whom had reported improvement during immunotherapy trials prior to evaluation at Mayo Clinic (Table 6, including 3 that would have met HE criteria had no other diagnosis been considered). None of 12 had objective improvement. In those patients, a diagnosis of autoimmune encephalopathy was initially entertained, but after subsequent evaluations, and considering the absence of response to immunotherapy, patients were eventually given an alternative diagnosis including neurodegenerative disorder (*n* = 6), FND, fibromyalgia, insomnia and depression, autism spectrum disorder, behavioural symptoms in patient with trisomy 21, and mast cell activation disorder (one each).

**Table 6:** Twelve patients ‘non-responders’—who received immunotherapy trial, with no improvement and were eventually diagnosed with non-autoimmune diagnosis

No/Age/ Sex	Onset <3m	Neurological presentation	Prior IT response	NPS abnormal	MRI abnormal	EEG abnormal	AE criteria	Final diagnosis
40 79 F	–	Memory loss, aphasia, apraxia, psychosis and seizures	–	+	Multiple lacunar strokes	Periodic sharp wave complexes	–	Probable Creutzfeldt–Jakob disease
41 48M	–	Cognitive impairment	–	+	Confluent T2-HI hemispheres and pons	NA	–	Frontotemporal dementia
43 52 F	–	Aphasia	–	NA	Temporal atrophy (L)	NA	–	Primary progressive aphasia
44 52 F	–	Aphasia and memory loss	+	+	–	Sharp waves, TIRDA	–	Primary progressive aphasia
45 53 F	–	Cognitive impairment, hallucinations and aphasia	+	+	–	Atypical spike and wave	–	Probable neurodegenerative disorder
46 71 F	+	Sub-acute confusion superimposed on chronic parkinsonism	+	NA	–	Diffuse slowing	HE	Probable neurodegenerative disorder
47 50M	–	Acute onset tremors and cognitive complaint	+	–	–	–	–	Functional disorder
48 43 F	–	Cognitive complaint, pain and fatigue	–	+ (Mild executive deficits)	–	Bitemporal slowing	–	Fibromyalgia
49 12M	–	Agitation, fixations (ASD)	+	+	–	–	–	ASD, behavioural
50 22F	+	Sub-acute change behaviour and cognition (trisomy 21)	+	NA	–	Diffuse slowing	HE	Trisomy 21, behavioural
51 22M	–	Cognitive impairment	+	–	–	–	–	Mast cell activation disorder
52 28F	+	Sub-acute insomnia, shaking spells and disorientation	NA	–	–	–	HE	Insomnia, spells and mood symptoms

+, yes; −, no; m, months; ASD, autism spectrum disorder; IT, immunotherapy; NPS, neuropsychological testing; NA, not available; T2-HI, T2-hyperintensity; L, left; AE, autoimmune encephalitis; HE, Hashimoto encephalopathy; TIRDA, temporal intermittent rhythmic delta activity.

Comparing the characteristics of these two groups, more patients in the responder group had an inflammatory CSF (38% versus 0% *P* = 0.02) (Table 7). Due to the sparse outcomes available, OR is not reported for the results of CSF. There were no statistically significant differences in other variables.

**Table 7:** CSF findings in patients with alternative diagnosis without clinical improvement after immunotherapy (non-responders) and patients with autoimmune CNS disorders who experienced improvement (responders)

Diagnostic test	Non-responders (*n* = 12)	Responders (*n* = 16)	Total (*n* = 28)	*P* value
CSF inflammatory^a^	0	6 (37.5)	6 (37.5)	**0.02**
WBCs, >5 cells/μl	0	4 (25)	4 (25)	0.11
Protein level, >50 mg/dl	5 (41.7)	9 (56.3)	14 (50)	0.7
Positive OCB	0/11	1/14 (7.1)	1/25 (4)	>0.99
Elevated IgG synthesis rate	0/11	1/14 (7.1)	1/25 (4)	>0.99

Categorical data is provided as number (percentage).

WBC, white blood cells; OCB, oligoclonal bands.

a CSF inflammatory: WBC, >5 cells/μl, positive OCBs, high IgG index or synthesis rate.

The median response to immunotherapy in epilepsy and encephalopathy 2 score was insensitive (though specific) for immunotherapy response in this largely neural IgG seronegative group; 5 (range, 0–10) among responders, and 2 (range, 0–5) among non-responders.

### Autoimmune CNS disorder relapses

After the initial immunotherapy trial at Mayo clinic, 11 responders, 9 of whom had reported relapses prior to our evaluation, initiated a steroid-sparing agent, including mycophenolate mofetil (*n* = 7), azathioprine (*n* = 3) and rituximab (*n* = 1). Two additional patients initiated azathioprine and methotrexate, respectively, after they experienced a relapse during follow-up. Follow-up data was available for 8 of these 13 patients who were treated with a steroid-sparing agent for a median duration of 2 years (range, 0.4–8), with a gradual steroid taper (either IV or oral) for a median duration of 8 months (range, 3–16).

At least 6 months of longitudinal data after treatment at Mayo Clinic was available for 11 responders (median 37 months; range, 13–108). In total, 6 of 11 (55%) had relapses: three while on a steroid-sparing agent only at 3, 10 and 36 months; one who had been on steroid-sparing agent for 10 months (stopped early because of upper respiratory infection), and relapsed 6 months after discontinuation and two who did not receive a steroid taper or maintenance immunosuppression after IVMTP treatment (at 6 weeks and 1 year, respectively).

Among three patients with neural-specific antibody defined disorders, only the GAD65 antibody-positive case had follow-up, and did not experience significant improvement of her seizure frequency after a trial of IVMTP and subsequent trial of IVIg.

Thirty-four patients reported side effects of immunotherapy (either prior to or after our evaluation), 13/39 (33%) in the autoimmune diagnosis group and 21/105 (20%) in the alternative diagnosis group.

## Discussion

Almost three-quarters of patients referred to our specialty practice as HE/SREAT in the context of thyroid autoimmunity left Mayo Clinic with an alternative non-autoimmune CNS diagnosis. The assignment of an autoimmune CNS diagnosis (usually autoimmune encephalopathy) to the remaining patients occurred on the basis of a thorough history and examination, and reliance on objective measurements. These include both measure of neurological dysfunction (cognitive testing, imaging and EEG) and CNS inflammation (encephalitis-specific antibody positivity, or elevations in WBC count, IgG index/synthesis rate or supernumerary OCB). Our findings validate the AE criteria proposed previously (Graus *et al.*, 2016). Uncertainty persisted in a small minority because of the unusual initial time course of symptoms, or seizure presentation. Follow-up testing of objective neurological dysfunction to discern improvement from pre-treatment baseline was useful in confirming the diagnosis in cases such as those, though more common treatment trials assisted in refuting an autoimmune diagnosis altogether.

TPO antibody values in the patients diagnosed with autoimmune CNS disorders were not significantly different to those in patients with an alternative clinical diagnosis, and the proportion of patients with very high TPO titres was similar in both groups. Patients with autoimmune CNS disorders were diagnosable, utilizing more specific neurological and immunologic metrics. Thyroid antibodies are serologic markers of autoimmune thyroiditis, and have little utility beyond that disease. TPO antibody detection frequency is ∼13% in healthy individuals (who are at risk for developing autoimmune thyroiditis), is more common among women and prevalence increases with age (27% of women >60 years old) (Hollowell *et al.*, 2002). Thyroid antibodies also co-exist in patients with other systemic autoimmune disorders (up to 50% prevalence in diabetes mellitus type 1, 45% in primary biliary cirrhosis and 18–26% in myasthenia gravis) (Nakamura *et al.*, 2008), in patients with other immune-mediated neurologic disorders such as multiple sclerosis, and AE with specific neural antibodies, with high titres (>200 IU/ml) in ∼8% of the patients. (Tuzun *et al.*, 2011; Mattozzi *et al.*, 2020). TPO antibodies are also found in 10% of patients with psychiatric admissions (affective disorders, schizophrenia, dementia, other psychosis and personality disorders) (Oomen *et al.*, 1996), up to 28% of patients with degenerative dementia (Kalmijn *et al.*, 2000) and 14% of patients with genetic cerebellar ataxias (Sivera *et al.*, 2012). Even markedly elevated thyroid antibodies have been found incidentally in patients with rapidly progressive dementias in which a non-immune aetiology was confirmed pathologically (Schott *et al.*, 2003).

Diagnostic testing findings in HE/SREAT have been previously reported as non-specific, with normal MRI or non-specific T2 signal abnormalities, elevated CSF protein level without elevated WBC count and diffuse background EEG slowing (Chong *et al.*, 2003; Castillo *et al.*, 2006; Ferracci and Carnevale, 2006; Laurent *et al.*, 2016). In our experience, the lack of a specific clinical syndrome and objective abnormalities on diagnostic testing may contribute to misdiagnosis in patients presenting with cognitive decline, if a comprehensive exclusion of other aetiologies is not pursued, and a diagnosis of autoimmunity is made based solely on the presence of elevated thyroid antibodies.

Neuronal antibodies were not systematically investigated in the previously reported series of patients diagnosed with HE/SREAT (Chong *et al.*, 2003; Ferracci and Carnevale, 2006; Olmez *et al.*, 2013; Laurent *et al.*, 2016). In our study, 3 of the 39 patients diagnosed with autoimmune CNS disorders were found to have neural antibody biomarkers (high titre GAD65, α-amino-3-hydroxy-5-methyl-4-isoxazolepropionic acid receptor and unclassified). Equal numbers of patients in both CNS autoimmune and alternative diagnosis groups were found to have low titre of neural antibodies detected by ELISA or immunoprecipitation assays, which are also prone to generating results of uncertain significance. These include low-titre GAD65 (Walikonis and Lennon, 1998; Muñoz-Lopetegi *et al.*, 2020), voltage-gated potassium channel (with negative leucine-rich glioma-inactivated 1 and contactin-associated protein-like 2 IgGs) (van Sonderen *et al.*, 2016a; Michael *et al.*, 2020), N-type and P/Q-type voltage-gated calcium channel (Zalewski *et al.*, 2016), ganglionic acetylcholine receptor (McKeon *et al.*, 2009) and striational antibodies (McKeon *et al.*, 2013).

All but three of our patients to whom we assigned autoimmune diagnoses were classifiable into one or other group (HE, limbic encephalitis and probable AE), utilizing the criteria proposed by Graus *et al.* (2016); sensitivity (92%) and specificity (100%). We encountered two additional cases of autoimmune dementia, where the history was that of cognitive decline without true encephalopathic delirium, and could have been mistaken for a neurodegenerative diagnosis. The young age of one, and the sub-acute, fluctuating course after more insidious symptoms in the other, prompted detailed investigations. The findings of CSF, EEG and MRI assisted in making autoimmune diagnoses. Both patients also responded to immunotherapy. In addition, consideration of alternative diagnoses, rather than a binary ‘it is either autoimmune or it is not’ is also critical to optimize use of the criteria for specificity, and good patient care, in general. In addition, one patient with thyroid autoimmunity and seizures, though without encephalopathy (in the context of high-titre GAD65 antibody) was also referred to us as a possible HE/SREAT case. In neurological autoimmunity in general, ‘outliers’ with a more restricted clinical phenotype that do not have a classical disease onset or phenotype may elude diagnosis.

Despite the lack of predictive value of thyroid antibody titres, a clinical history of a co-existing autoimmune disorder was more common among those with an autoimmune CNS diagnosis. Other clinical clues that were supportive were sub-acute onset of encephalopathy, the presence of seizures, stroke-like episodes, aphasia and ataxia. Stroke-like episodes have been reported as HE-typical (Brain *et al.*, 1966; Shaw *et al.*, 1991; Chong *et al.*, 2003). In our autoimmune CNS disorder cohort, stroke-like episodes appeared more common among those with biochemical evidence of sub-clinical thyroid dysfunction (seven out of eight cases). Future studies should attempt to identify more specific biomarkers for this sub-group. Depressive symptoms, anxiety, chronic pain and the absence of objective abnormalities were more common in patients with an alternative diagnosis. The most critical factor to make a diagnosis of autoimmune CNS disorder was the demonstration of objective findings such as abnormalities in CSF analysis, brain MRI and/or PET, and EEG in 25 out of 39 cases. In two additional cases, the finding of specific neural antibodies confirmed the autoimmune CNS diagnosis. The remaining 12 cases presented with sub-acute encephalopathy, met HE criteria (Graus *et al.*, 2016) and had reported improvement with immunotherapy, demonstrated objectively after new immunotherapy trial at Mayo Clinic in three cases. A 6-week trial of immune therapy with pre- and post-objective neurological testing often assists us in determining the likelihood of clinically meaningful benefit from longer-term treatment.

Although we did not find remarkable differences in the statistical analysis of the neuropsychological testing scores, it is important to note that by the time of our evaluation, many patients had already received an immunotherapy trial, which could have led to an improvement in the scores in the autoimmune CNS cases, thus masking some abnormalities. It is worth noting that the delayed recall score in both Kokmen STMS and Auditory Verbal Learning Test was significantly different between the autoimmune CNS disorder and non-neurological groups. This suggests that delayed recall impairment is an additional clue that may help identify autoimmune CNS patients. In addition to the test scores, neuropsychologist interpretation of abnormalities in the clinical context was critical, particularly in patients with aetiologically challenging cognitive complaints. Functional neurological disorders, mood disorders, sleep disorders, untreated chronic pain, untreated sleep apnoea and polypharmacy were common. A recent systematic review found that around a quarter of patients presenting to memory clinics with cognitive symptoms were diagnosed with subjective cognitive impairment, pseudo-dementia, functional cognitive disorder or a primary psychiatric disorder, and not degenerative brain disease or other medical cause (McWhirter *et al.*, 2020).

Subjective clinical improvements, without documentation of objective changes in examination, had been reported by one-third of patients ultimately given alternative diagnoses. In our experience, corticosteroids at high doses cause a non-specific endocrinologic peak-dose ‘steroid-buzz’ characterized by increased energy and mental acuity, which wanes rapidly between doses. This pattern contrasts with the immune-suppressive effects of steroids, resulting in gradual recovery in autoimmune encephalopathy, which typically starts no earlier than after several days of continuous treatment. Reliance on reported subjective improvements alone led to over-diagnosis of a steroid-responsive encephalopathy. Among patients in the autoimmune CNS group who had follow-up, objective neurological improvements were documented in bedside cognitive testing, neuropsychometric testing, EEG or imaging. CSF inflammatory abnormalities appear to have value for both diagnosis and treatment–response prediction. After comprehensive exclusion of alternative aetiologies, an immunotherapy trial may be considered in patients presenting with sub-acute encephalopathy. Ongoing surveillance for emergence of alternative aetiologies (which may also be steroid responsive, such as lymphoma) is also required in those cases. A predictive model of response to immunotherapy has been developed for encephalopathy, the response to immunotherapy in epilepsy and encephalopathy 2. A score of ≥7 has been reported to have a sensitivity of 96% and specificity of 86% (Dubey *et al.*, 2018). This score was not informative in this largely neural IgG-negative cohort (2 points are given for encephalitis-specific IgG positivity).

Most patients with suspected autoimmune CNS disorders received IVMP as the first-line acute therapy. IVIg and plasmapheresis were also used in some cases. This was typically followed by a slow taper over several months. Many patients had reported relapses prior to our evaluation, though we surmise from our experience that those patients likely initially received steroid courses of inadequate dose and duration. Maintenance immunosuppressive therapy was initiated, mainly with oral agents such as mycophenolate mofetil and azathioprine, with subsequent slow taper of steroids. During follow-up at Mayo Clinic, 6 out of 11 patients with at least 6 months of longitudinal data available reported relapses, though largely in the context of short treatment duration. Others have reported a relapse rate after initial treatment in previous case series of patients diagnosed with HE/SREAT varying from 16 to 60% (Castillo *et al.*, 2006; Ferracci and Carnevale, 2006; Laurent *et al.*, 2016). Although only a small number of patients had enough longitudinal data available, it is noteworthy that the frequency of relapses in this case series was higher than the relapse rate reported for leucine-rich glioma-inactivated 1 and anti-*N*-methyl-d-aspartate receptor encephalitis (20–30%) (Titulaer *et al.*, 2013; Arino *et al.*, 2016; van Sonderen *et al.*, 2016b).

This study was not powered to analyse long-term outcomes in detail. However, many patients with autoimmune encephalopathy have residual symptoms such as long-term cognitive difficulties (van Sonderen *et al.*, 2016b; de Bruijn *et al.*, 2018), personality change, depression, headache and sleep disorders (Ariño *et al.*, 2020), long after the initial presentation, which deserve further research.

In the initial SREAT case series of 20 patients, the objective data supporting a diagnosis of autoimmune CNS disorder was limited (Castillo *et al.*, 2006). For example, the severity of EEG slowing was not mentioned, and when this is mild and diffuse, it is non-specific and sometimes secondary to central acting medication effects (Marcuse, 2016), rather than secondary to an autoimmune encephalopathy. Although 17 out of 20 patients in that series had elevated CSF protein (non-specific in our cohort), other more specific inflammatory abnormalities were rare (elevated WBC count in 25%, OCB in 5% and elevated IgG synthesis rate in 10%). A more recent case series had similar limitations of available objective data supportive of autoimmune diagnoses (Litmeier *et al.*, 2016). Although the diagnosis of SREAT was defined by response to steroid treatment in both [62.5% (20/32) patients with sub-acute encephalopathy associated with autoimmune thyroiditis in the Castillo *et al.*, 2006 series], neither study presented documentation of objective measures of response to immunotherapy (Castillo *et al.*, 2006; Litmeier *et al.*, 2016).

We acknowledge several limitations of this study. Given the retrospective nature of the study, there was not a uniform collection of data, given some variation in evaluations undertaken. Over time our practice has become more uniform. Referral bias may have influenced the characteristics of these patients. Neural antibodies were not investigated in CSF in 46 patients, which may have limited our ability to detect disorders such as anti-*N*-methyl-d-aspartate receptor encephalitis (Gresa-Arribas *et al.*, 2014) and autoimmune glial fibrillary acidic protein astrocytopathy (Flanagan *et al.*, 2017), though none of the patients presented with those phenotypes. As mentioned previously, many of the patients had received immunotherapy prior to our evaluation, which may have affected the results of some of the investigations. Longitudinal improvements in cognitive scores may also have been subject to learning or practice bias effects.

## Conclusion

We conclude that thyroid antibodies have served their time as diagnostic biomarkers in autoimmune encephalopathy well, but their role in the evaluation of autoimmune encephalopathy is likely redundant at this point, and certainly less specific than a clinical history of autoimmune disease and neural-specific antibodies. Our experience indicates that a diagnosis of HE/SREAT is often given to patients presenting with cognitive symptoms and a variety of neurological and non-neurological complaints, in the setting of elevated thyroid antibodies in serum without objective cognitive abnormalities. The utility of testing for thyroid antibodies seems questionable in the modern era which has brought availability of validated clinical criteria and advanced neuroimmunologic diagnostics. Over diagnosis of autoimmune encephalopathy brings undesired consequences such as iatrogenic harm, cost of unnecessary immunosuppressive therapies and delayed diagnosis of the correct neurological disorder. As always, test results need to be interpreted in the context of detailed clinical history and examination.

## Supplementary material

Supplementary material is available at *Brain Communications* online.

## Competing interests

S.J.P. is a named inventor on filed patents that relate to functional AQP4/NMO-IgG assays and NMO-IgG as a cancer marker. He has patent pendings for LUZP4, KLHL11, Septin 5 and MAP1B IgGs as markers of neurological autoimmunity and para-neoplastic disorders. He has consulted for Alexion and Medimmune. He has received research support from Grifols, Medimmune and Alexion. All compensation for consulting activities is paid directly to Mayo Clinic. S.J.P. has a patent pending for KLHL11-IgG as a marker of neurological autoimmunity. D.D. has received research support from Center of Multiple Sclerosis and Autoimmune Neurology, Center for Clinical and Translational Science and Grifols pharmaceuticals. He has consulted for UCB and Astellas pharmaceuticals. All compensation for consulting activities is paid directly to Mayo Clinic. Dr Dubey has a patent pending for LUZP4-IgG and KLHL11-IgG as markers of testicular cancer and neurological autoimmunity. E.P.F. is a site principal investigator in a randomized placebo-controlled clinical trial of Inebilizumab (A CD19 inhibitor) in neuromyelitis optica spectrum disorders funded by MedImmune/Viela Bio. He receives no personal compensation and just receives reimbursement for the research activities related to the trial. A.Z. has a patent pending for PDE10A-IgG as a marker of neurological autoimmunity. A.M. has patents pending for LUZP4, KLHL11, PDE10A, Septin-5, -7 and MAP1B IgGs as markers of neurological autoimmunity and para-neoplastic disorders. He has received research support from Grifols, Medimmune and Euroimmun but has not received personal compensation. And the other authors have no disclosures.

## Supplementary Material

fcaa233_Supplementary_Data

## Notes

See Rodenbeck and Clardy (https://doi.org/10.1093/braincomms/fcab030) for a scientific commentary on this article.

## Abbreviations

AE =: autoimmune encephalopathy
APE =: antibody prevalence in epilepsy
ASD =: Autism spectrum disorder
AZT =: azathioprine
B =: bilateral
CYC =: cyclophosphamide
F =: female
FND =: functional neurological disorder
GAD65 =: glutamic acid decarboxylase 65-kD isoform
GPEDs =: generalized periodic epileptiform discharges
HE =: Hashimoto encephalopathy
Hippo =: hippocampus
IT =: immunotherapy
IVIg =: intravenous immunoglobulin
IVMTP =: intravenous methylprednisolone
L =: left
LE =: limbic encephalitis
M =: male
m =: month
MMF =: mofetil mycophenolate
mRs =: modified Rankin score
NA =: not available
NPS =: neuropsychological testing
PLEX =: plasma exchange
Pred =: prednisone
Prot =: protein
R =: right
RITE =: response to immunotherapy in epilepsy
RTX =: rituximab
SE =: side effects
S-L =: stroke-like
SOE =: symptom onset excluded
SPECT =: single-photon emission-computerized tomography
SREAT =: steroid-responsive encephalopathy associated with autoimmune thyroiditis
STMS =: Kokmen short test of mental status
TIRDA =: temporal intermittent rhythmic delta activity
TPO =: thyoperoxidase
T2-HI =: T2-hyperintensity
y =: year
w =: week
WBC =: white blood cells
