# Supplementary material for: Brain dysfunction and thyroid antibodies: autoimmune diagnosis and misdiagnosis
Source: Brain Commun. 2021 Jan 5;3(2):fcaa233. doi: 10.1093/braincomms/fcaa233 (PMC8152924; doi:10.1093/braincomms/fcaa233)
Supplement: fcaa233_Supplementary_Data [file fcaa233_supplementary_data.docx]

**Supplementary Methods**

**Neural antibody testing**

Serum and CSF samples were screened by standardized mouse tissue-based indirect immunofluorescence assays (IFA) for IgGs with the following specificities: adaptor protein-3B2 (AP3B2), α-amino-3-hydroxy-5-methyl-4-isoxazole propionic acid receptor (AMPA-R), amphiphysin, anti-glial nuclear antibody (AGNA)-1, anti-neuronal nuclear antibody (ANNA)-1, ANNA- 2, ANNA-3, aquaporin-4, collapsin response-mediator protein 5 (CRMP5), contactin-associated protein 2 (CASPR2), contactin-1, dipeptidyl-peptidase-like protein 6 (DPPX), ɣaminobutyric acid B receptor (GABAB-R), glial fibrillary acidic protein (GFAP), glutamic acid decarboxylase-65 isoform (GAD65), GTPase regulator associated with focal adhesion kinase pp125 (GRAF-1), inositol trisphosphate receptor (ITPR-1), leucine-rich glioma inactivated protein-1(LGI-1), metabotropic glutamate receptor (mGluR) 1, mGluR5, neurochondrin, neuronal intermediate filament, N-methyl-D-aspartic acid receptor (NMDA-R), IgLON5, Purkinje cell cytoplasmic autoantibody (PCA) type 1, PCA type 2 and PCA type Tr, septins 5 and 7, and as yet unclassified neural-specific autoantibodies.

Cell-based assays employing human embryonic kidney (HEK) 293 cells transfected with appropriate expression plasmids were used to detect or confirm IgGs specific for AMPA-R, CASPR2, DPPX, GABAB-R, LGI1, NMDA-R.

Radioimmunoprecipitation assays were performed to detect or confirm autoantibodies specific for neuronal voltage-gated (Kv1) potassium channel-complexes (VGKCs), GAD65, P/Q type voltage-gated calcium channels (VGCC), N-type VGCC, nicotinic acetylcholine receptors (muscle-type and ganglionic-type ).

Enzyme-linked immunosorbent assay (ELISA) was performed to detect striational antibodies.

**Cognitive testing**

1. Screening test:

Kokmen short test of mental status (STMS), which assesses and scores points for orientation (8), attention (7), learning (4), calculation (4), abstraction (3), construction (4), information (4), and recall (4). The maximum score is 38 points (Kokmen *et al.*, 1991).

B) Neuropsychological test results in the following cognitive domains were collected:

1) Intellectual function (Wechsler Adult Intelligence Scale [WAIS] perceptual organization score which uses visual-spatial appreciation and problem-solving tasks to quantitate nonverbal reasoning ability; and verbal comprehension score which tests general knowledge, vocabulary and abstraction to quantitate verbal reasoning ability) (Wechsler, 1999).

2) Premorbid intelligence (Wide Range Achievement Test–Revision 3 reading subtest) (Wilkinson, 1993).

3) Learning and memory (Auditory Verbal Learning Test [AVLT] which examines learning and memory for data sets that are too large to manage in working memory) (Rey, 1970).

4) Language (Controlled Oral Word Association Test (Benton AL, 1994), Boston Naming Test (Kaplan E, 1978), and Category Fluency Test (Lucas *et al.*, 1998).

5) Executive function (Trail-Making Test [TMT] which measures the time taken for the participant to connect the dots of 25 numbers scattered on a screen, and B in which the participant connects 25 numbers and 25 letters, alternating between the two) (Reitan, 1992).

6) Overall cognition with the Dementia Rating Scale (Hughes *et al.*, 1982).

**References for cognitive testing:**

Benton AL HK, Sivan AB. Multilingual Aphasia Examination. 3rd ed ed. San Antonio, TX: Psychological Corporation; 1994

Hughes CP, Berg L, Danziger WL, Coben LA, Martin RL. A new clinical scale for the staging of dementia. Br J Psychiatry 1982; 140: 566-572.

Kaplan E GH, Weintraub S. The Boston Naming Test. Philadelphia, PA: Lea & Febiger; 1978.

Kokmen E, Smith GE, Petersen RC, Tangalos E, Ivnik RC. The short test of mental status. Correlations with standardized psychometric testing. Arch Neurol 1991; 48(7): 725-728.

Lucas JA, Ivnik RJ, Smith GE, Bohac DL, Tangalos EG, Graff-Radford NR*, et al.* Mayo's older Americans normative studies: category fluency norms. J Clin Exp Neuropsychol 1998; 20(2): 194-200.

Reitan R. Trail Making Test: Manual for Administration and Scoring. Tucson, AZ: Reitan Neuropsychology Laboratory; 1992.

Rey A. L' examen clinique en psychologie. Paris, France: Presses Universitaries de France; 1970.

Wechsler D. Wechsler Abbreviated Scale of Intelligence (WASI). San Antonio, TX: Harcourt Assessment; 1999.

Wilkinson GS. Wide Range Achievement Test-Revision 3 (WRAT-3). Wilmington, DE: Jastak Association; 1993.

**Supplementary Table 1. Summary of variables associated with an autoimmune CNS diagnosis by univariate Firth’s penalized likelihood regression analysis**

|  | **Autoimmune**  **CNS disorder (n=39)** | **Alternative**  **diagnosis (n=105)** | **Total**  **(n=144)** | **OR**  **(95% CI)** | **p value*** |
| --- | --- | --- | --- | --- | --- |
| Subacute onset | 32 (82.1) | 29 (27.6) | 61 (42.4) | 11.24  (4.52, 27.9) | **<0.001** |
| Coexisting autoimmune disorder | 13 (33.3) | 17 (16.2) | 30 (20.8) | 2.58  (1.11, 5.97) | **0.04** |
| Seizures | 10 (25.6) | 8 (7.6) | 18 (12.5) | 4.08  (1.5, 11.1) | **0.008** |
| Stroke-like episodes | 8 (20.5) | 5 (4.8) | 13 (9) | 4.93  (1.55, 15.6) | **0.007** |
| Language deficit | 11 (28.2) | 13 (12.4) | 24 (16.7) | 2.76  (1.12, 6.8) | **0.04** |
| Ataxia | 6 (15.4) | 4 (3.8) | 10 (6.9) | 4.38  (1.22, 15.7) | **0.02** |
| Low vitamin B12 | 6/26(23.1) | 1/70(1.4) | 7/96(7.3) | 14.69  (2.29, 94.4) | **0.001** |
| MRI abnormalities suggesting AE | 8/39 (20.5) | 4/104 (3.8) | 12/143 (8.4) | 6.03  (1.78, 20.4) | **0.003** |
| Abnormal EEG | 14/33 (42.4) | 14/85 (16.5) | 28/118 (23.7) | 3.67  (1.50, 8.96) | **0.007** |
| CSF inflammatory | 20/39 (51.3) | 20/88 (22.7) | 40/127 (31.5) | 3.51  (1.58, 7.82) | **0.002** |
| CSF WBC >5 cells/mcL | 9/39 (23.1) | 1/88 (1.1) | 10/127 (7.9) | 18.17  (3.06, 108) | **<0.001** |

Categorical data provided as number (percentage). Bold values denote statistically significant results (p<0.05).

*p values from Fisher test

**Supplementary Table 2. Comparison of the Kokmen scores in the autoimmune CNS disorders group vs neurodegenerative group & autoimmune group vs non-neurological disorder**

| **Kokmen score** | **Median of differences** | **Bonferroni-adjusted CI** | **p-value** |
| --- | --- | --- | --- |
| Autoimmune CNS group vs neurodegenerative | | | |
| **Total** | -11.0 | (-21, -4) | <.001 |
| **Orientation** | -2 | ( -5, 0) | <.001 |
| **Learning** | -1 | ( -2, -1) | <.001 |
| **Calculation** | -2 | ( -3, 0) | 0.04 |
| **Abstraction** | -1 | ( -1, 0) | <.001 |
| **Construction** | -2 | ( -3, -1) | <.001 |
| **Information** | -1 | ( -2, 0) | <.001 |
| **Recall** | -1 | ( -2, 0) | 0.03 |
| Autoimmune CNS group vs non-neurological disorder | | | |
| **Recall** | 1 | (0, 2) | 0.03 |

(Only statistically significant differences shown)

**Supplementary Table 3. Comparison of the neuropsychological testing scores in the autoimmune CNS disorders group vs neurodegenerative group & autoimmune encephalopathy group vs non-neurological disorder**

| **Test score** | **Median of differences** | **Bonferroni-adjusted CI** | **p-value** |
| --- | --- | --- | --- |
| Autoimmune CNS group vs neurodegenerative group | | | |
| **WAIS perceptual organization** | -29 | (-59 ,-12) | 0.02 |
| **TMT B** | 95 | ( 4 ,251) | 0.03 |
| Autoimmune CNS group vs non-neurological disorder | | | |
| **AVLT delayed recall** | 5 | (0, 9) | 0.02 |

(Only statistically significant differences shown)

Abbreviations: WAIS (Wechsler Adult Intelligence Scale, TMT (Trail-Making Test), AVLT (Auditory Verbal Learning Test)

**Supplementary Table 4. Neural antibodies found in serum in patients with autoimmune CNS disorders & non-autoimmune diagnosis**

| **Neural autoantibody** | **Autoimmune CNS disorder**  **(n=14)** | **Non-autoimmune diagnosis**  **(n=21)** |
| --- | --- | --- |
| **AMPA-R**  **Number of patients** | 1 | 0 |
| **GAD65**  **Number of patients**  **High titer**  **Low titer median (range)** | 8 (1 high and 7 low)  294  0.1 (0.06 - 0.15) | 10  0.23 (0.1 - 4.88) |
| **VGKC (LGI-1/CASPR2 negative)**  **Number of patients**  **Median titer (range)** | 3  0.11 (0.08 - 0.13) | 1  0.18 |
| **VGCC N-Type**  **Number of patients**  **Median titer (range)** | 2  0.11 (0.08 - 0.14) | 2  0.445 (0.27 - 0.62) |
| **P/Q VGCC**  **Number of patients**  **Median titer (range)** | 1  0.07 | 3  0.09 (0.04 - 0.15) |
| **Striational**  **Number of patients**  **Titer** | 1  1:240 | 2  1:240, 1:480 |
| **AchR ganglionic**  **Number of patients**  **Median titer (range)** | 1  0.11 | 5  0.1 (0.04 - 0.275) |
| **AchR binding**  **Number of patients**  **Median titer (range)** | 2  0.205 (0.19 - 0.22) | 0 |

Antibody titers measured in nmol/L. Reference ranges: GAD65 ≤ 0.02 nmol/L, VGKC ≤ 0.02 nmol/L, VGCC N-Type ≤ 0.03 nmol/L, P/Q VGCC ≤ 0.02 nmol/L, striational ≤ 1:60, Ach ganglionic ≤ 0.02 nmol/L, AchR binding ≤ 0.02 nmol/L

Abbreviations: AMPA-R = α-amino-3-hydroxy-5-methyl-4-isoxazolepropionic acid receptor, GAD65 = glutamic acid decarboxylase 65-kilodalton isoform, VGKC= voltage gated potassium channel, LGI-1= leucine-rich glioma inactivated 1, CASPR2= contactin-associated protein 2, VGCC = voltage gated calcium channel, AchR= acetylcholine receptor

**Supplementary Figure 1. Neuroimaging findings before & after immunotherapy in two of the patients with autoimmune encephalopathy diagnosis**


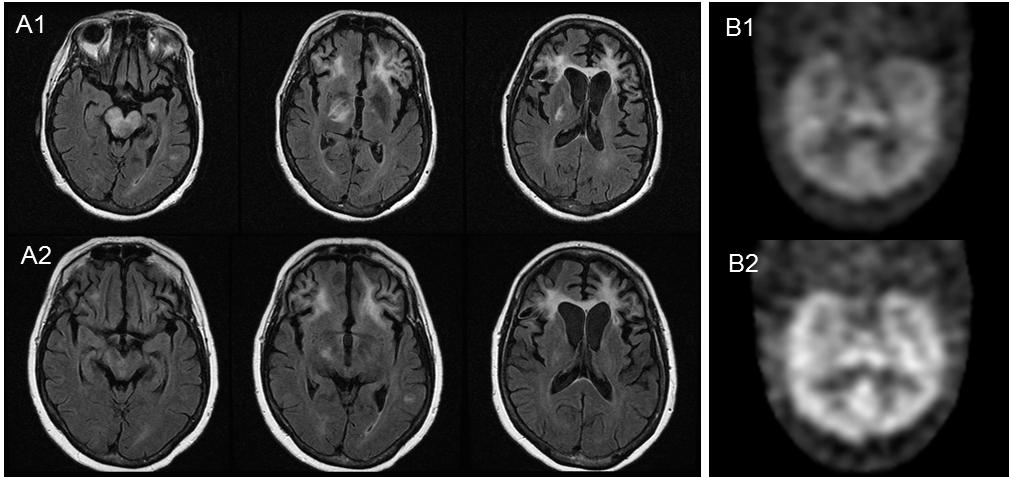


Magnetic resonance imaging corresponding to patient 2 in table 5: axial fluid-attenuation inversion recovery (FLAIR) sequence shows abnormal T2 hyperintensity within the bilateral frontal lobes (residual after prior episode) and new T2 hyperintensity of the right internal capsule, and midbrain (A1), improved after treatment with intravenous methylprednisolone (A2). Single-photon emission computed tomographic imaging corresponding to patient 1 in table 5: diffused frontal, temporal and parietal decreased uptake (B1), with improvement after treatment with intravenous methylprednisolone (B2).
